# Discrete image recovery via stochastic resonance in optically induced photonic lattices

**DOI:** 10.1038/s41598-019-48313-y

**Published:** 2019-08-14

**Authors:** Yongbin Zhang, Hongjun Liu, Nan Huang, Zhaolu Wang

**Affiliations:** 1 State Key Laboratory of Transient Optics and Photonics, Xi’an Institute of Optics and Precision Mechanics, Chinese Academy of Sciences, Xi’an, 710119 China; 2 Collaborative Innovation Center of Extreme Optics, Shanxi University, Taiyuan, 030006 China; 3 University of Chinese Academy of Sciences, Beijing, 100084 China

**Keywords:** Nonlinear optics, Nonlinear optics

## Abstract

We demonstrate numerically the discrete image recovery via stochastic resonance in optically induced photonic lattices. The underlying signals are regularly reinforced at the expense of scattering noise with the interplay of the periodic potentials and the self-focusing nonlinearity. We founded that the energy redistribution tends to be periodic and the signal reinforcement is promoted with the help of periodic potentials. The lattice intensity levels, applied voltages, and correlation lengths are important parameters to influence the recovery effects. The dynamic nonlinear evolution including intensity and power spectrum is modeled according to the two-dimensional quasi-particle motion model. Our results suggest a potential technology to detect the noisy images.

## Introduction

Imaging through scattering media, such as smokes, tissues, and cloudy water, is one of the fundamental problems in optics. Random collisions with particles disturb light transmission and destroy the image structure. The underlying signals are often overwhelmed by highly scattering noise. Conventional optical imaging technologies, such as spatial filtering, polarization discrimination, and time gating, intuitively improve image quality by rejecting the detrimental noise^1–3^. These technologies are limited because some valuable signals accompanying with noise are inevitably prevented. Additionally, it is impossible to filter the noise without energy loss.

The received images are mainly degraded by chaotic noise when passing through a scattering medium. In particular, noise is not always harmful to signals. In some nonlinear systems, weak signals can be enhanced with the assistance of moderate noise, which is described as stochastic resonance (SR)^4^. The phenomenon of SR occurs in many fields, such as biology, electrology, and optics^5–7^. Many works focus on one-dimensional signals rather than two-dimensional images. Furthermore, in the field of nonlinear image recovery, SR was first developed and exploited by Dylov *et al*. to recover the noise-hidden images propagating in a photorefractive crystal^8,9^. The coherent signals are enhanced at the expense of incoherent noise by seeding modulation instability under the self-focusing nonlinearity. Then Han *et al*. applied the SR to reconstruct the nanosecond-pulse noisy images theoretically and experimentally because pulse lasers are widely used in target detection^10,11^. Afterward, Dylov *et al*. extended the SR to recover the diffused images, while the reconstructed images are in a low contrast^12^.

The type of SR has been observed in a homogeneous nonlinear medium, but not reported in optically induced photonic lattices. Wave propagation in periodic lattices is quite different from that occurring in a homogeneous medium^13–15^. The localization tends to be periodic and better due to tunnelling in the lattices. The discrete self-focusing effects appear and hold a great potential to inhibit wave diffusion.

In this paper, we demonstrate numerically the discrete image recovery via SR in optically induced photonic lattices. The underlying signals, as a source of instability, are regularly enhanced at the expense of scattering noise under the discrete self-focusing nonlinearity. We founded that mode coupling and signal enhancement are promoted by periodic localization due to tunnelling in the lattices. The dynamic nonlinear evolution including intensity and power spectrum is simulated according to the two-dimensional quasi-particle motion model. The reconstructed images are obtained in the form of periodic spots by optimizing lattice intensity levels and applied voltages. Our results indicate a potential method for recovering scattering images in various imaging applications.

## Results

### Framework of discrete image recovery via SR

**Figure 1:**
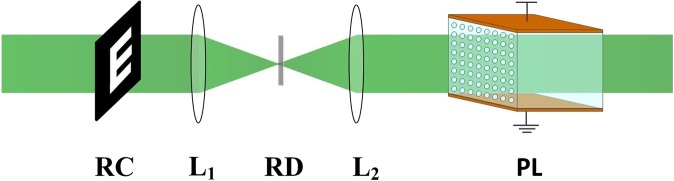
Framework of discrete image recovery via SR. RC, resolution chart; L, lens; RD, rotating diffuser; PL, optically induced photonic lattices.

Figure 1 shows the framework of discrete image recovery via SR in our simulation. The continuous expanded laser beam first passes through a resolution chart to generate a coherent binary image. The rotating diffuser is considered to scatter the coherent signals and produce the diffused images (~0.14 W/cm^2^). Then the scattering signals are incident into the optically induced photonic lattices for achieving the discrete image recovery. An applied voltage across the crystalline *c* axis is used to control the nonlinearity. The two-dimensional photonic lattices can be created through an optical induction technique^13^. The periodic potentials are induced by interfering two pairs of ordinarily polarized plane waves in a photorefractive crystal (SBN:75, 8 × 8 × 10 mm^3^, *r*_33_ = 1340 pm/V, and *r*_13_ = 67 pm/V).

### Numerical analysis of discrete image recovery via SR

**Figure 2:**
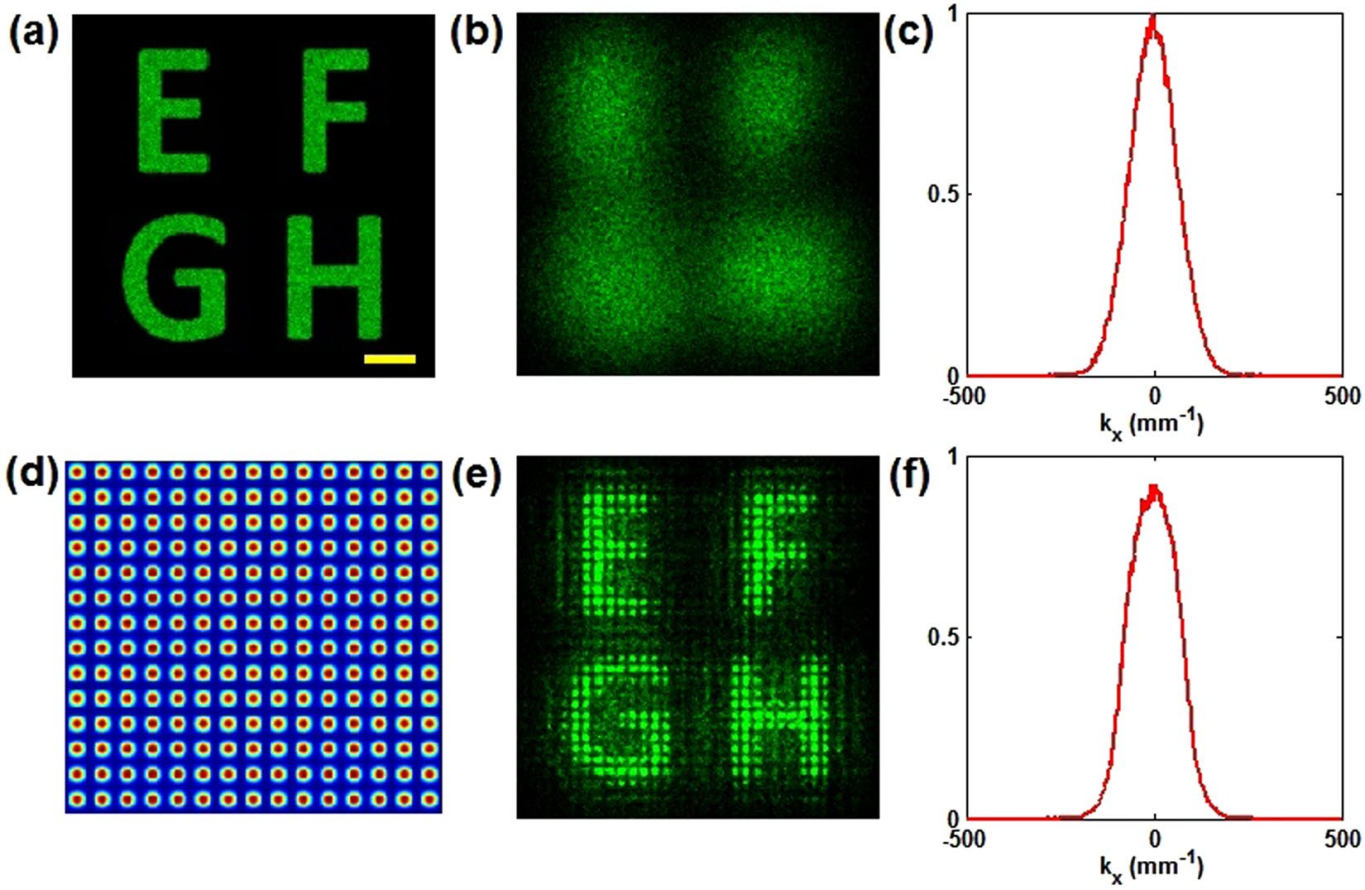
(**a**) Original image, (**b**) scattering image and (**c**) its normalized power spectrum, (**e**) nonlinear recovered image and (**f**) its normalized power spectrum. (**d**) Periodic potentials. Yellow scale bar, 50 μm.

The simulation results of the discrete image recovery via SR are presented in this section. The nonlinear process is analyzed by treating the light as a large number of quasi-particles and tracing their transmission in a nonlinear medium. The motion of each quasi-particle is restrained by the Hamiltonian equations. The intuitive process is that the positions and momentums of quasi-particles are continuously updated due to the refractive index change until the light exits from the crystal. More specially, the results are obtained by iteratively solving the Hamiltonian equations with 100 steps across 10 mm^16–18^. Each iteration consists of the following four steps: (1) calculate the intensity distribution; (2) calculate the photo-induced refractive index; (3) solve the Hamiltonian equations for each photon; (4) update the positions and momentums of photons. The intensity and momentum (power spectrum) distributions in the simulation process are both statistically obtained through a particle-in-cell method. As shown in Fig. 2(a), an original image is very clear without being scattered. Once scattered, the image is overwhelmed by chaotic noise, as shown in Fig. 2(b). Its corresponding power spectrum shown in Fig. 2(c) is considered as a Gaussian type. Figure 2(d) shows the periodic potentials with the lattice spacing of 11 μm. Figure 2(e) shows a recovered spot-forming image under the discrete self-focusing nonlinearity. It is seen that the diffusion is inhibited and the submerged image becomes obviously visible. The energy redistribution tends to be periodic. The corresponding power spectrum is displayed in Fig. 2(f). The profile becomes shorter and wider than that in Fig. 2(c). This is because mode coupling occurs and causes the spatially frequency transfer from low to high^19^.

**Figure 3:**
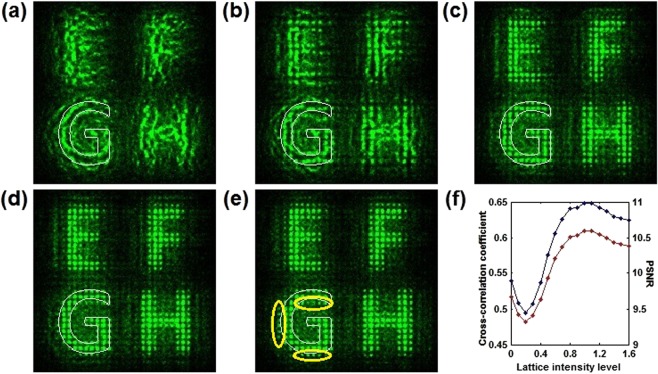
Nonlinear recovered images with different lattice intensity levels. (**a**) 0, (**b**) 0.2, (**c**) 0.6, (**d**) 1, (**e**) 1.4. The white closed curve represents the outline of the original image. The yellow elliptical curve highlights incoherent noise solitons outside the outline of the original image under the strong discrete self-focusing nonlinearity. (**f**) Cross-correlation coefficient (blue) and PSNR (red) as a function of lattice intensity levels.

The nonlinear recovered results are dependent on lattice intensity levels (the ratio between the peak intensities of the lattice beam and the signal beam), applied voltages, and correlation lengths of scattering signals. As shown in Fig. 3(a–e), we first consider the nonlinear recovered results as a function of lattice intensity levels. The applied voltage and the correlation length are fixed as 1500 V and 0.1 mm, respectively. The lattice intensity levels represent the modulation depth of periodic potentials. As shown in Fig. 3(a), without the effect of periodic potentials, the self-focusing nonlinearity leads to the signal reinforcement by seeding modulation instability^9,12^. However, the recovered image consists of randomly distributed patterns caused by modulation instability, namely optical Langmuir turbulence, which disorders the image structure. As shown in Fig. 3(b), when the lattice intensity level is low, the weak periodic potentials are considered as a perturbation and have no promotion for image recovery. As the lattice intensity levels are increased further, the image quality is rapidly improved and the images consisting of periodic spots are displayed in Fig. 3(c,d). The best recovered image is shown in Fig. 3(d), when the lattice intensity level is 1. As shown in Fig. 3(d), the bright periodic patterns (Δ*n* = 3.4 × 10^−4^) reflect the structure of the original image. The localization tends to be periodic and better due to the periodic potentials in the lattices, which is helpful to inhibit the wave diffusion and promote the signal reinforcement^20–22^. However, when the level is higher than 1, the strong discrete self-focusing nonlinearity causes the appearance of incoherent noise solitons (marked with yellow elliptical curves) outside the outline of the original image “G” (shown in Fig. 3(e))^23^. The profile of the spot-forming image is broadened, which decreases the image quality. The broadening effect also exists in the other three spot-forming images “E”, “F”, and “H”. Furthermore, the performance metrics, such as the cross-correlation coefficient and the peak signal-to-noise ratio (PSNR), are used to quantitatively evaluate the nonlinear recovered results^17^. As shown in Fig. 3(f), the two metric curves first decrease, then increase, and finally decrease. It is seen that when the level is lower than the threshold of 0.2, the image quality is decreased due to the periodic perturbation. Once the level is higher than the threshold, the quality grows rapidly until the level reaches the second threshold of 1. As the level is increased further, the quality begins to decrease because the strong discrete self-focusing nonlinearity causes the generation of incoherent noise solitons outside the original image.

**Figure 4:**
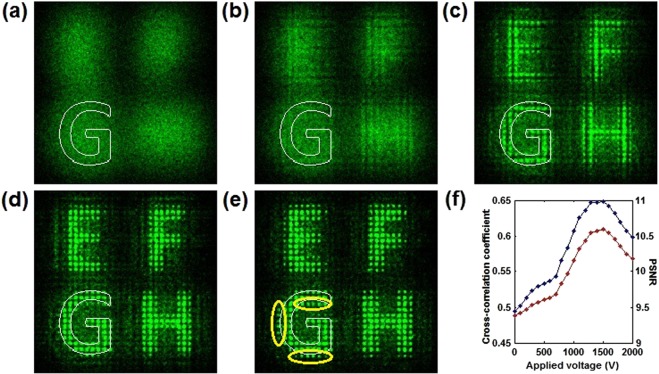
Nonlinear recovered images with different applied voltages. (**a**) 0 V, (**b**) 500 V, (**c**) 1000 V, (**d**) 1500 V, (**e**) 2000 V. (**f**) Cross-correlation coefficient (blue) and PSNR (red) as a function of applied voltages.

Next, we examine the nonlinear recovered images with different applied voltages. The lattice intensity level and the correlation length are fixed as 1 and 0.1 mm, respectively. The signal beam and the lattice beam are simultaneously incident into the nonlinear media. As shown in Fig. 4(a), the scattering image submerged by chaotic noise is completely invisible without an applied voltage. As shown in Fig. 4(b), when the voltage is low, the signals have no obvious contrast improvement. This is because weak nonlinearity insufficiently prevents wave diffusion, but only causes the modulation of power spectrum^19^. Once the voltage is high enough, the visibility of the signals is rapidly improved and the images in the form of periodic spots are clearly observed in Fig. 4(c,d). The underlying signals are regularly reinforced at the expense of scattering noise under the discrete self-focusing nonlinearity. The periodic potentials tends to promote the signal reinforcement and achieve the periodic energy redistribution. As the voltage is increased further, the strong discrete self-focusing nonlinearity causes the appearance of incoherent noise solitons outside the original image, which decreases the image quality. In Fig. 4(f), the metric curves clearly show the variation of image quality as a function of applied voltages. The energy is conserved in the whole nonlinear process. The reinforcement of signals indicates the occurrence of the directional energy transfer from the chaos noise to signals, which is an indicator of SR^8,9^. Moreover, the appearance of an optimal image recovery effect at different applied voltages reveals the characteristic signature of SR.

**Figure 5:**
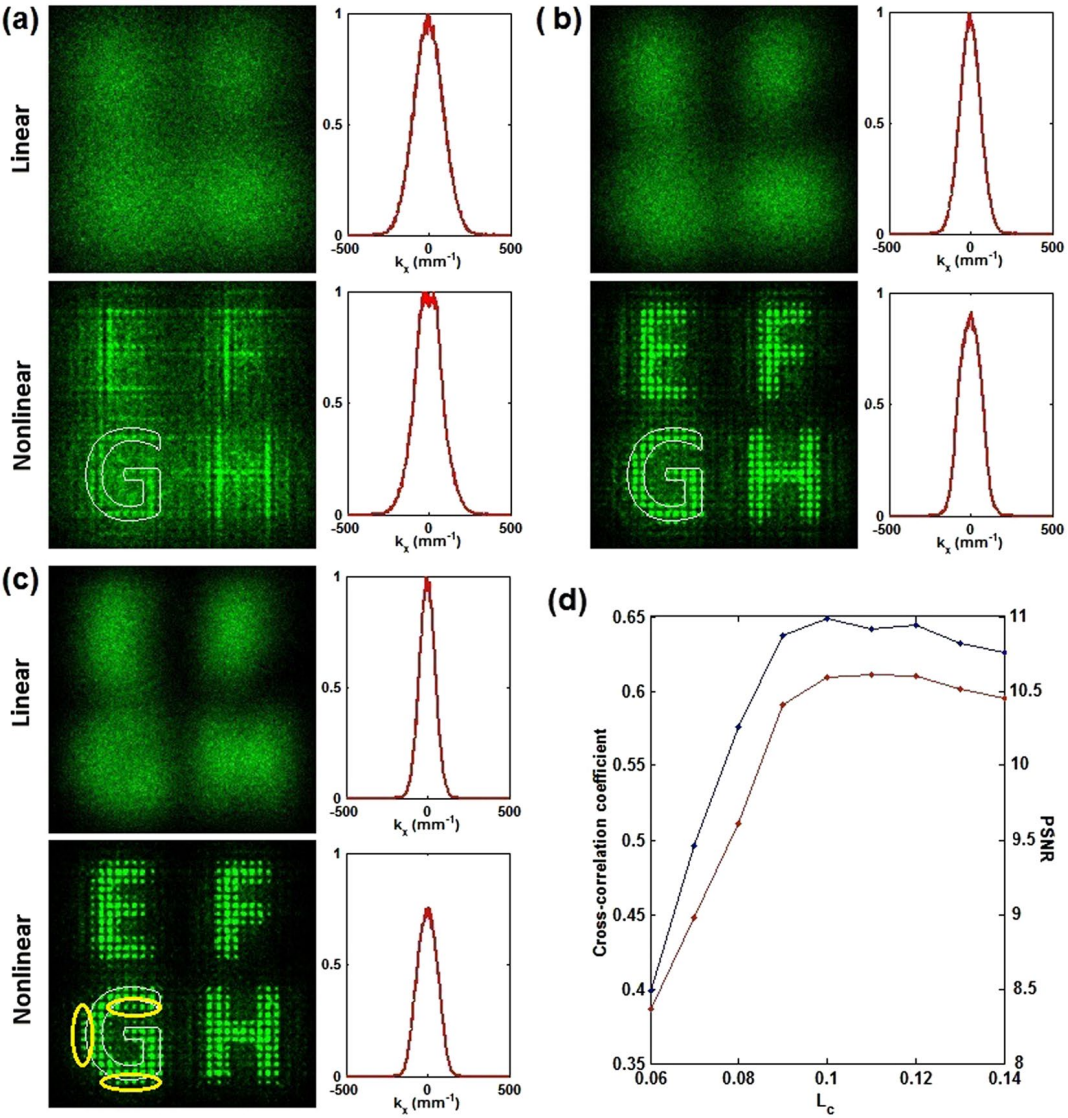
Linear (nonlinear) output images (normalized power spectrum) with different correlation lengths. (**a**) 0.07 mm, (**b**) 0.1 mm, (**c**) 0.13 mm. (**f**) Cross-correlation coefficient (blue) and PSNR (red) as a function of correlation lengths.

Figure 5 shows the linear (nonlinear) output images (normalized power spectrum) with different correlation lengths. The applied voltage and the lattice intensity level are fixed as 1500 V and 1, respectively. As shown in Fig. 5(a–c), the intensity distribution of linear output images becomes more and more concentrated with the increase of correlation lengths. The correlation length reflects the diffusion degree of scattering images. The long correlation length means the scattering signals are slightly diffused. The corresponding linear power spectrum of Gaussian type becomes narrower and narrower. Then it is seen that all the nonlinear recovered images have a better visibility compared with the linear output images. This means SR occurs and the underlying signals are enhanced by extracting energies from chaotic scattering noise under the discrete self-focusing nonlinearity. Moreover, it is seen that the corresponding power spectrum becomes shorter and wider than original spectrum. The reason is that the underlying signals are a source of instability. Mode coupling causes the spatially frequency transfer from low to high under the nonlinear condition. Among the recovered images, the recovered image shown in Fig. 5(a) is surrounded by much noise and in a low contrast. This is because it is difficult to assemble the photons with large scattering angles through the discrete self-focusing nonlinearity. As shown in Fig. 5(c), the nonlinear recovered image has the better visibility. This indicates that the scattering signals with smaller angle diffusion are easy to be recovered at the same nonlinear condition. However, the strong intensity modulation causes the generation of incoherent noise solitons outside the original image, which slightly degrades the image. Finally, the recovered result showed in Fig. 5(b) has the highest value of performance metrics. Correspondingly, the ratio of the lattice spacing to the correlation length is 0.11. The detail metric curves as a function of correlation lengths are shown in Fig. 5(d).

**Figure 6:**
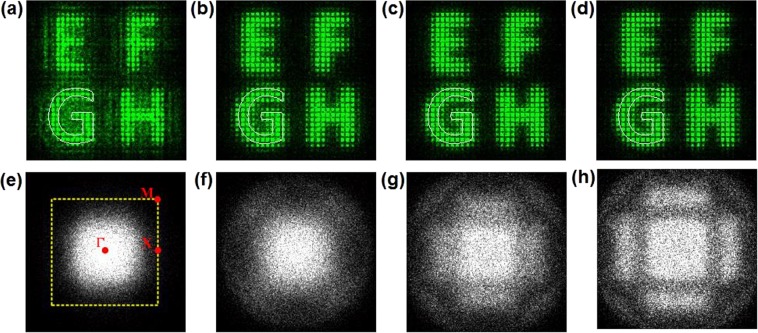
Nonlinear output images (spatial power spectrum) with high lattice intensity levels. (**a**–**d**), nonlinear output images; (**e**–**h**), spatial power spectrum. Lattice intensity levels: (**a**,**e**) 1, (**b**,**f**) 10, (**c**,**g**) 20, (**d**,**h**) 30.

Remarkablely, the number of full band gaps depends on the depth of potentials in optically induced optical lattices^24,25^. Only the potentials that is deep enough support the complete band gap. It is best to analyze the lattice properties by observing the spatial power spectrum of the transmitted light beam. Figure 6 shows the nonlinear output images and the corresponding spatial power spectrum with high lattice intensity levels. The applied voltage and the correlation length are fixed as 1500 V and 0.1 mm, respectively. The energy distribution of the enhanced signals tend to be periodic with the assistance of weak periodic potentials when the lattice intensity level is low, as shown in Fig. 6(a). For the weak periodic potentials, the different bands overlap with each other and no complete band gap exists according to ref.^23^. The power spectrum of the scattering light covers a large portion of the first Brillouin zone, as shown in Fig. 6(e). As the high lattice intensity levels are set, the periodic potentials dominate the nonlinear process and almost completely constrain the light propagation. The profile of the spot-forming images is broadened, which decreases the image quality. When the level is higher than 20, the band gap opens up between the ***k*** vectors of the first two Brillouin zones. The lattice-dominated nonlinearity leads to the generation of random-phase lattice solitons, as shown in Fig. 6(d). The power spectrum shown in Fig. 6(h) takes on the square symmetry of the lattices and has the obvious separated humps, with four peaks located in the normal diffraction regions of the first two Brillouin zones. It is seen that the discrete image recovery is realized through the combined effect of self-focusing nonlinearity and the periodic potentials rather than through the independently dominated nonlinearity.

## Discussion

The results suggest that the weak periodic potentials promote the periodic localization of the light beam. The neighbouring potentials are not isolated, but allow the energy exchange between the adjacent tunnels. The energy distribution of enhanced signals tends to be periodic and better under the discrete self-focusing nonlinearity in optically induced photonic lattices.

In conclusion, the discrete image recovery via SR has been numerically demonstrated in optically induced photonic lattices. The underlying signals are regularly enhanced at the expense of scattering noise under the discrete self-focusing nonlinearity. The localization tends to be periodic and the signal enhancement is promoted with the help of the periodic potentials. The recovered images consisting of periodic spots are clearly observed with the suitable parameters. The two-dimensional quasi-particle motion model is used to simulate the dynamic nonlinear evolution. The results indicate a potential method for noisy image detection.

## Method

The discrete image recovery via SR is numerically studied according to the framework shown in Fig. 1. The scattering signals incident into the nonlinear media are partially coherent. Several theoretical approaches, such as the mutual coherence function approach, the self-consistent multimode theory, the coherent density method, Hamiltonian ray tracing, and the quasi-particle motion model have been proposed to describe the partially coherent beam propagation in a nonlinear medium^16,17,26,27^. Here, we use the previously created quasi-particle motion model to analyze the nonlinear propagation of statistical light^17^. The motion of quasi-particles obeys the Hamiltonian equations which describe the response of light to refractive index change. In detail, Hamiltonian equations derived from the radiation transfer equation based on the Liouville theorem are given by^16,17^

1 $${d}{\boldsymbol{r}}=\frac{{\boldsymbol{k}}}{{{k}}_{0}}\cdot {dz},$$

2 $${d}{\boldsymbol{k}}=\frac{\partial \langle {\rm{\Delta }}{n}\rangle }{\partial {\boldsymbol{r}}}\cdot {dz},$$

where ***r***(*r*_*x*_, *r*_*y*_) and ***k***(*k*_*x*_, *k*_*y*_) are the position and momentum vectors perpendicular to the propagation direction z, *k*_0_ = 2π*n*_0_/*λ* is the wave number in a medium with the base index of refraction *n*_0_. Δ*n* is the nonlinear index change created by the photorefractive screening nonlinearity^28^:

3 $${\rm{\Delta }}n=\frac{1}{2}{n}_{0}^{3}{r}_{33}\frac{V}{d}\frac{{I}_{L}+{I}_{S}}{1+{I}_{L}+{I}_{S}},$$

where *r*_33_ is the electro-optic coefficient, *V* is the applied voltage across the crystalline *c* axis, *d* is the thickness of the crystal, *I*_*L*_ and *I*_*S*_ represent the light intensities of the signal beam and the lattice beam, respectively. The lattice intensities are described as *I*_*L*_ = *I*_*L*,*P*_*cos*^2^(π*x*/*D*)cos^2^(π*y*/*D*), where *D* represents the lattice spacing^24^. The momentum distribution of the scattering signals is a Gaussian type [*f*(***k***) = *I*_0_/(2πΔ*k*^2^)exp(−(*k*_*x*_^2^ + *k*_*y*_^2^)/(2Δ*k*^2^))], where Δ*k* = 2π/*l*_*c*_ is the spectral spread with correlation length *l*_*c*_.
